# Implementation and contribution of temperature screening and handwashing practice at points of entry for COVID-19 pandemic response in a humanitarian crisis setting

**DOI:** 10.11604/pamj.2022.43.127.28171

**Published:** 2022-11-07

**Authors:** Lawali Mahaman Rabiou, Batouré Oumarou, Blanche-Philomene Melanga Anya, Mutenda Sheria Kaya, Tambwe Didier, Biey Joseph Nsiari-Muzeyi, Patrick Katoto, Charles Shey Wiysonge

**Affiliations:** 1 World Health Organization, Quartier Plateau, Avenue Mohamed VI 1204, Niamey, Niger,; 2 World Health Organization, Sub-Regional Office for West Africa, Ouagadougou, Burkina Faso,; 3 Cochrane South Africa, South African Medical Research Council, Francie van Zijl Drive, Parow Valley 7501, Cape Town, South Africa,; 4 Centre for Tropical Medicine and Global Health, Faculty of Medicine, Catholic University of Bukavu, Bukavu, Democratic Republic of Congo,; 5 Department of Global Health, Faculty of Medicine and Health Sciences, Stellenbosch University, Francie van Zijl Drive, Tygerberg 7505, Cape Town, South Africa,; 6 School of Public Health and Family Medicine, University of Cape Town, Anzio Road, Observatory 7935, Cape Town, South Africa,; 7 HIV and Other Infectious Diseases Research Unit, South African Medical Research Council, Durban, South Africa

**Keywords:** SARS-CoV-2, humanitarian emergency, behaviours change communication, Diffa, Niger

## Abstract

**Introduction:**

over the last decade, insecurity in the Lake Chad Basin has triggered an unprecedented humanitarian crisis in the Niger´s Diffa Region with a significant population movement. In this humanitarian setting, we reviewed the implementation process and the contribution of temperature screening and handwashing practice at points of entry as part of non-pharmaceutical interventions against COVID-19.

**Methods:**

in Diffa, border officers were trained on the fundamentals of infection prevention and control in relation to COVID-19 readiness and response and a 14-day district response team was constituted. To examine the significance of the implementation process of temperature screening and handwashing practices at points of entry, we conducted a secondary analysis of data submitted by the six health districts of the Diffa Region between March and July 2020.

**Results:**

travellers screened for fever ranged from 10,499 (in March 2020) to 62,441 (in April 2020) with the health districts of Diffa (mean: standard error of the mean: 25,999: 9,220) and of Bosso (mean: standard error of the mean: 30.4: 19.1) accounting for the most and the least of activities during the entire period, respectively. Overall, 125/169,475 travellers presented fever and were effectively quarantined. Only the Ngourti Health District reported travellers who declined handwashing (54/169,475); this was during the first three months of the first wave of the COVID-19 pandemic.

**Conclusion:**

we have documented a successful implementation of measures related to temperature screening with some unsubstantial denial of handwashing. Given the importance of border traffic due to insecurity in the Diffa Region, maintaining temperature screening and handwashing in this humanitarian setting is necessary but requires coordinated actions of all stakeholders involved in the region.

## Introduction

The Lake Chad Region, which includes Nigeria, Niger, Chad, and Cameroon has experienced violent attacks by armed groups of Boko Haram. The relentless attacks perpetrated for more than a decade in this region are characterized by multiple dynamics of instability and a state of emergency [1]. To avoid areas of insecurity, people have moved in search of a more secure and comparatively safe environment. The scale of this state of insecurity was marked by an unprecedented humanitarian crisis in the Diffa Region of Niger. The latter host Nigerian refugees fleeing terrorist attacks in Northern Nigeria since 2013. According to the United Nations High Commissioner for Refugees (UNHCR), since the first strikes on Niger at the beginning of 2015, the situation has escalated dramatically [2]. Consequently, the state of insecurity has contributed to the weakness of the health system in the region. In one hand, some health facilities have closed and on other hand, the remaining have been overwhelmed by the high demand for health care, not just from indigenous communities but also from displaced and refugee populations.

In addition to this crisis, on the 11^th^ March, 2020, the World Health Organisation (WHO) has announced a new pandemic of COVID-19 following SARS-CoV-2 infection on March 11, 2020 [3]. As of May, Niger has reported 955 positive cases, including 65 deaths while, the Diffa Region has reported only 19 suspicious cases of which seven were positive [4]. However, the risk of importation of the disease in Diffa, was considered as very high due to its proximity with the Zinder Region, which reported 137 positive cases, the Bornou State in Nigeria, which reported 30 cases and Chad, which also reported several cases. COVID-19 contamination can occur directly after contact with infected individuals (asymptomatic as well symptomatic or indirectly after contact with contaminated materials (organic as well inorganic)) [3]. Screening for temperature [5] and proper hand hygiene are important to prevent the spread of SARS-CoV-2 infections [6]. Notification of warnings from travellers suspected of developing a new coronavirus infection might lead to quarantine, initial management, and referral of ill travellers for better care and contact tracing. Considering the high migration flow in Diffa following the humanitarian crisis, implementing a full of set checklist for screening at points of entry might just be hard to implement and maintain but useful to alleviate the health system.

We hypothesised that temperature screening in conjunction with regular handwashing was implemented successfully and hence contributed to the effort to curb the spread of COVID-19 in this humanitarian area.

## Methods

**Study design, setting, and population:** we conducted a secondary analysis of data submitted by the six health districts of the Diffa Region from March 2020 to July 2020. The Diffa Region is in the extreme South-East of Niger between 10° 30' and 15° 35' east and 13° 04' and 18° 00' north. It spans 156,906 km^2^ and borders the territory of Agadez to the north, Chad to the east, Nigeria to the south and Zinder to the west and has a total of four functional points of entry. As of June 2020, the UNHCR [2] registered biometrically 224,416 displaced persons in the country (66,811 households), including 125,754 Nigerian refugees, 65,769 internally displaced persons (IDPs), 30,797 returnees (Niger nationals who had emigrated but had returned to Niger) and 2,097 asylum seekers. The UNHCR census has been stopped in March due to insecurity and COVID-19. However, the latest governmental estimates released in May 2020 reported 265,617 displaced persons.

**Interventions under evaluation:** temperature screening and hand washing at point of entry across the major cities of Diffa, a humanitarian setting.

**Implementation strategy:** the Diffa Region has six health districts: Diffa, Bosso, Ngourti, N´guigmi, Maine Soroa and Goudoumaria. Boarder officers have been trained on the fundamental of infection and prevention controls in relation to COVID-19 readiness and response. While training considered the main component such as social distancing, mask wearing, a special attention with technical demonstrations was put on correct temperature screening using infrared thermometers and hand washing following the World Health Organization (WHO) guidelines. Poster translated in local languages were also placed across points of entry. Several nurses have also joined the team and necessary materials such as thermometers and handwashing tools built by local contractors were supplied by the national COVID-19 response team. If travellers were permitted to refuse hand washing, temperature screening was set to be mandatory. For the travellers to be able to comply with this process, the approach focused on strengthening the knowledge of each traveller on handwashing by a team of volunteers at the points of entry in a large city.

**Figure 1:**
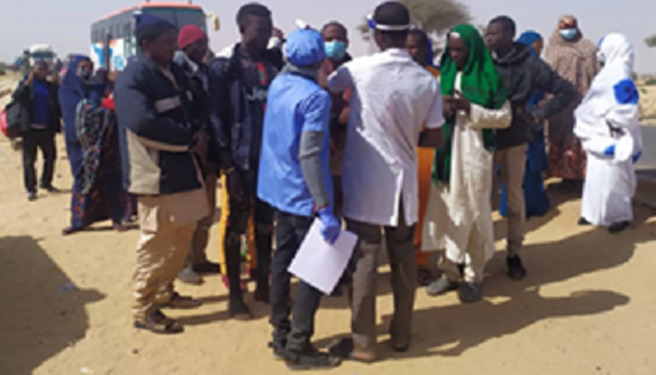
travellers arriving at a point of entry in Diffa Region, Niger, April 2020; a pair of trained health workers educating passengers on temperature control and handwashing practice directly when they get off the bus

**Monitoring of the implementation strategy:** a front worker team included two health workers and a driver to ensure the daily implementation of the temperature control combined with handwashing (Figure 1).

**Case management during implementation:** travellers showing temperature above 37.5°C were asked to sit down, waiting for 10 minutes before repeating the screening. If the temperature stayed >37.5°C, they were accompanied by the district response team and requested to not stay in the screening area. A 14-day district response team was constituted to ensure the management of suspected cases.

**Variables of interest to ensure success of the programme:** to ensure the program's success, we examined the adherence of travellers across Diffa's major cities to the implemented intervention, counting the number of travellers successfully checked for fever and agreeing to wash their hands.

**Data management and analysis:** the data were extracted from district´ registries using an Excel spreadsheet (Microsoft Office 2016), and GraphPad Prism (V8.2) was used to characterize the measures of tendency (mean and standard deviation or proportion/frequency) and to create the visual representation.

## Results

**Figure 2:**
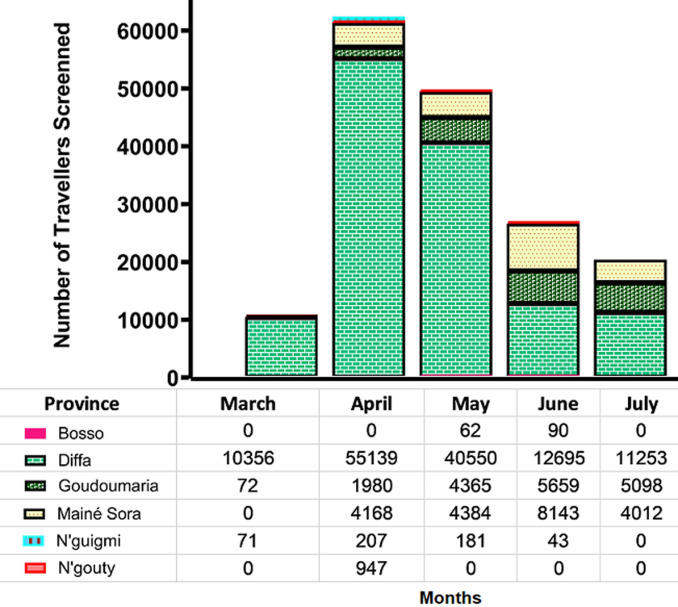
number of travellers crossing the ports of entry and successfully screened for temperature across six health districts in Diffa, Niger, from March to July 2020

**Count of travellers traversing the Diffa border from March 2020 to July 2020:** Figure 2 depicts the number of travellers screened at the point of entry across six health districts in Diffa from March to July 2020. With the procedures put in place, all the travellers traversing the points of entry across the major cities of Diffa were successfully screened for fever. The number ranged from 10,499 in March to 62,441 in April with the health districts of Diffa (mean, standard error of mean (SEM): 25999: 9220) and of Bosso (mean: SEM: 30.4: 19.1) accounting for the most and the less of activities during the entire period, respectively.

**Figure 3:**
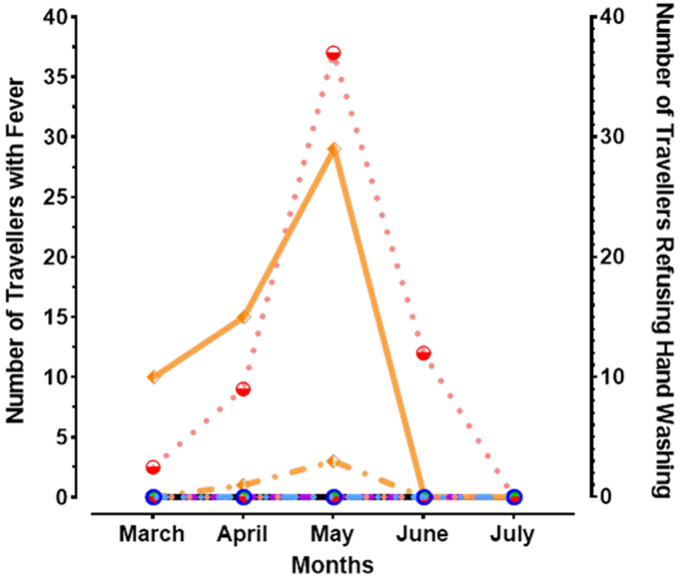
average number of travellers with fever after screening (incomplete lines) and with refusal of hand washing practice (complete line) at ports of entry by health districts and by months in Diffa, Niger, from March to July 2020

**Attitudes of travellers toward fever screening and hand washing at the point of entry in Diffa:** overall, 125/169,475 travellers were found with fever and effectively quarantined. Of them, 121 were screened in Diffa Health District and the remaining in 4 in N'gourti Health District. While the most of cases were reported during the month of May, no traveller with fever was reported in July. Only the N'gourti Health District has registered travellers who declined to wash their hands before the temperature was tested (54/169,475) with the highest number also observed in May. With the evolution of the pandemic, no health district has reported travellers who declined to wash their hands before in June and July (Figure 3).

## Discussion

We have documented the implementation process and the contribution of temperature screening and handwashing at points of entry in a conflict zone. We found a successful implementation of measure related to temperature screening with some unsubstantial denial of handwashing mostly reported in the Ngourti Health District during the first three months of the first wave of COVID-19 pandemic. The number of travellers screened above normal temperatures has agreed with the first pick of COVID-19 in Niger also observed between April-May 2020. All these passengers were successfully accompanied by our 14-days district team for further assessment.

It is currently known that temperature is one of the cardinal symptoms even though not specific of the early manifestation of SARS-CoV-2 infection [7,8]. Temperature regulation at ports of entry level has also been recommended, both on arrival and departure, for patients with severe acute respiratory syndrome (SARS), Ebola virus disease, H1N1, seasonal influenza, or dengue fever. These have been coupled with the traveller self-reporting of the disease, visual examination and the background of the previous visited areas and countries. The capacity to perform temperature tests will detect febrile persons and can take early steps like references for additional testing or guidance on home treatment [9]. In this way it is possible to proactively classify the disorder such that potentially ill individuals hold tight social distances, abstain from interaction with others and wear a mask.

However, it is currently known that asymptomatic transmission of COVID-19 contacts is possible and consequently, temperature screening my not be effective alone to rule out COVID-19 among travellers [8].

An individual who is infected with SARS-CoV-2 is likely to have contaminated hands and to spread the pathogen direct or via surfaces and objects, whether symptomatic and asymptomatic (fomites) [6]. The efficacy of daily handwashing on reducing viral infection as well childhood mortality has been largely studied [10,11]. Hands hygiene is one of the pillars of COVID-19 response in Niger. In comparison to influenza A virus, the 9-hour survival of SARS-CoV-2 on human skin may increase the risk of contact transmission and sub-consequently accelerating its spread in the community [12]. This is particularly important in Diffa where the culture also favours mass gathering during traditional ceremonies such funeral, initiation etc. During a pandemic, washing hands regularly using chlorination or an adequate amount of soap, rubbing the hands together to create friction, and rinsing under running water is cost-effective in such humanitarian area [13]. In 2019, 2.4 billion people globally lack access to soap and water hand washing mostly located in Africa with extremely poor access in rural and urban communities in the countries that have low income [6,14]. Strikingly, access to water and soap in facilities is associated with the double in odds of handwashing before food touch [6,15].

**Funding:** the implementation of this activity was supported by the State of Niger with the support of his partners.

## Conclusion

Temperature screening and handwashing for travellers at ports of entry in Diffa, a humanitarian setting, were implemented with success and helped to the community's COVID-19 response. Though temperature screening might not be accurate to rule out COVID-19 cases, when combined with strategically located handwashing stations and other non-pharmaceutical interventions such as physical distancing and proper usage of masks will help to improve messages for the group for COVID-19 readiness.

### What is known about this topic

- Handwashing and temperature screening are useful for COVID-19 infection control;
- Migration is a major risk factor for the spreading of COVID-19 infection.

### What this study adds

- Handwashing and temperature screening at points of entry are feasible in a humanitarian setting;
- Handwashing and temperature screening might improve messages for the group for COVID-19 readiness when sustained among travellers.
